# Retention of Full Dentures*Liberal abstract of paper published in the December *Dental Review*.

**Published:** 1919-01

**Authors:** Rupert E. Hall


### RETENTION OF FULL DENTURES*

BY RUPERT E. HALL, D.D.S.

The requirements of the construction are, that the base of the denture should cover and be accurately adapted to the entire useable surface of the jaw. It should have

---

* Liberal abstract of paper published in the December *Dental Review.*

added to it a periphery with border surface continuous with that of the base of the denture. The surface of such border should be extended upon, and be adapted to the flexible peripheral tissues, so that there is created a seal and valve-like action between the flexible peripheral tissues and the surface of the periphery to preclude the ingress of air under the base of the denture, and resist or prevent partial or complete dislodgement through the indirectly applied resisting atmospheric force, should displacement occur.

Preventing the ingress of air between the surface of the base of the denture and the tissues of the jaw at the time of partial displacement, by sealing the space occurring between the base and jaw without admitting the air, forms thereby simultaneously a partial vacuum.

The *tidal* or momentary partial vacuum created between the base of the denture and the adapted tissues of the jaw is therefore manifested only when the resistive forces of the cohesion of the molecules of the interposed saliva are overcome and displacement of the denture actually occurs. Since the resultant atmospheric pressure is the direct force holding the denture against partial and complete dislodgement when forces displace the structure. forming a relative partial vacuum, it is obvious that any force that creates the space, degree of vacuity and resultant atmospheric pressure, increases automatically, thereby, resistance of the denture to further partial or complete dislodgement. The degree of vacuity of the space is in direct ratio to the volume of the vacuum, owing to the fact that the sealed periphery precludes the ingress of air and the increasing space between the base and the jaw still accommodates the same quantity of air. Boyle's Law governing the relationship between the pressure and volume of gases under a constant temperature covers this point and is as follows:

"Pressure of a given mass of gas varies inversely as the volume of the space within which it is confined."

That is, if the volume of space existing under the denture consists of 1 c.c. at a pressure of one atmosphere, when increased to 2 c.c. of volume the pressure according to this law would be one-half of an atmosphere.

Since we know the relation or state defined as adaptation does not exist when an artificial denture is first introduced into the mouth, and until adaptation evolves by wear, permitting the tissues, as they do, to fill in and shape themselves to conformity and apposition with the surface of the base, we also know that during this introductory period, so to speak, while the tissues are adjusting themselves, establishing adaptation or the relation termed basal seat, partially evacuated space or spaces exist between the base and jaw about areas not in sufficiently close apposition to establish adhesion. Therefore, in this connection, it is conceded that atmospheric pressure is an aiding force retaining a denture conjointly with adhesion and cohesion. Also, does it solve the perplexing puzzle of why many dentures lose their fit—so called? Nature responds to the negative pressure of evacuation, the tissues fill in and obliterate the space or spaces and the force of the atmosphere becomes nil and the denture is retained in the position of basal seat by adhesion and cohesion only.

Extent of adapted tissue surface then, determines the relative extent of the respective forces exerted by adhesion and cohesion retaining the artificial denture.

Peripheral extension and adaptation for a seal and valve-like action with the flexible peripheral tissues, sealing space occurring between the base and jaw created by displacement of the denture, preventing the ingress of air, forming a relatively increasing evacuated space indirectly applying the force of the atmosphere thereby, aids in preventing partial and complete dislodgement of the artificial denture should displacement occur.

DENTURE ADAPTATION

The ideal construction then, for the maximum utilization of the forces of adhesion, cohesion and atmospheric pressure for the retention of an artificial denture against displacement, partial and complete dislodgement, is to have the base cover all practicable surface of the jaw, and to add to the base a periphery, extended upon and adapted to the flexible peripheral tissues sealing the peripheral border with the flexible peripheral tissues.

Credit for the idea of constructing and establishing such relations between the base and its periphery, with the flexible peripheral tissues, should be given Dr. W. V-B Ames, of Chicago, for it was he who first conceived of their importance and in 1885 (*Independent Practitioner,* July) demonstrated their principles. Others notable in early appreciation and use of these principles, were the Greene Brothers, of Missouri, and special admiration and appreciation should be held for their untiring, constant and persistent labors for their adoption. Credit belongs to these men, so we understand, for the correctible compound method. Also the excellent work of Mr. Samuel G. Supplee, of New York, on the technic in the use of modeling compound should not be overlooked. Through his work and efforts our knowledge and technic in impression taking has been greatly improved.

The method of denture construction to be illustrated and described permits, it is believed, of an infinitely greater surface tissue adaptation and wider range of movements of the denture without causing displacement and unsealing or breaking of the peripheral valve seal than is secured in less accurate methods of construction of the old method where peripheral adaptation and valve seal are entirely absent and the edges of the denture permit the ingress of air and dislodgement of the restoration.

### IMPRESSION TAKING

The basic essential in the taking of a perfect impression for full and complete utilization of the jaw and flexible peripheral tissues desired to be utilized for the adaptation and retention of an artificial denture is a tray suited to the case. It is my belief that the only accurate and satisfactory way to procure a suitable tray, is to construct with a modeling compound a special tray for each individual case.

### IMPRESSION MATERIAL

Plaster of Paris, mixed to the correct consistency, is far more yieldable and adaptable than any other material with which we are familiar, and by the aid of a correctly formed individual tray, may be handled with such control that any desired form of impression may be secured. The particular feature of ''post-damming'' or adaptation, under pressure upon the flexible tissues of the soft palate, however, seems best accomplished with modeling compound.

### INDIVIDUAL TRAYS

For the construction of individual trays, we use the S. S. White Impression-Tray Compound, which was suggested and prepared for the particular purpose of making individual trays quickly, efficiently and economically. It is jet black in color to make it readily distinguishable; has a high melting point to assure, when set or hardened, ample rigidity against distortion in removal from the metal tray and subsequent shaping and handling.

It is necessary in employing the impression tray compound to have a few regular trays of suitable forms and sizes. Select a tray of the proper shape for the case, but somewhat larger than you would ordinarily use. Fill the tray with the compound, softened in hot water. Pass the exposed surface of the compound over a bunsen or alcohol flame to remove inequalities in the surface and give it a glaze. Plunge into hot water to wet the surface and prevent sticking to the tissues, and as soon as it cools to a bearable degree insert in the mouth and secure a compound impression in the regular way. In a short time it can be removed from the mouth and placed in cold water to harden.

Remove the impression from the metal tray, and with a sharp knife, trim away the excess compound approximating the peripheral outline and contour of the proposed tray. (Approximating the contour of the proposed restoration in the tray should be credited to Dr. M. M. House, of Indianapolis, Ind.)

### TRIMMING THE UPPER TRAY

Beginning at the labial margin, the tray is trimmed thin at the labial frenum, and the frenum allowed liberal relief. Passing on to the region formerly occupied by the cuspid teeth on either side, the tray is given a gradual fullness or prominence, restoring the cuspid eminences. The tray should be as high and full in this region as may be required to lift or displace the tissues for retention of the proposed denture and restoration of disturbed facial contour—the idea being to accentuate—build up the jaw ridge, increasing its area, and make in the finished impression the desired facial restoration.

Passing posteriorly from the cuspid eminences or about midway between the cuspid eminences and the tuberosities, we find the malar process of the maxilla, which registers a downward curve in the compound impression. The process is thinly covered with tissue and disposed at an unfavorable angle to permit of much vertical movement or bearing of the denture in this region and undue pressure of the same should be avoided.

Moving posteriorly of the malar process, a well defined cavity, as a rule, is found, and offers extremely valuable area for peripheral adaptation and denture retention. This space may be called the buccal cavity, and defined as the cavity formed or bound by the malar process, the check, the angle of the mouth and the tuberosity. It is indeed amazing how little this valuable space is utilized, and on the other hand quite astonishing how extensively it may be utilized.

In forming the tray for this space, allow it to go well up into the cavity, filling it buccally as well as vertically, preferring, however, to accentuate or favor vertical height rather than buccal fullness. Next, outline and trim the posterior or palatial border of the tray. The outline of the tray should approximate that of the junction of the hard with the soft palate. Its length, however, should extend well upon the soft palate, the exact length of which will be determined in a later operation. Finally, cut out the tray relieving locks about undercuts and points of impingement upon soft, flabby ridge tissues allowing them to hang freely in the tray.

Construction of dentures for upper jaws not requiring facial restoration or permitting presence of base and periphery upon the tissues in the labial region of the jaw and flexible peripheral tissues, do not permit peripheral adaptation in the buccal areas under pressure upon the flexible peripheral tissues. Construction and adaptation of periphery upon the flexible peripheral tissues in the buccal areas are desirable, but care should be exercised in preventing pressure, the positive force thus created by displacing the tissues would react against retention of the denture and would not, in the absence of periphery and peripheral valve seal in the labial region, be met with and overcome by atmospheric pressure by the forming of an emergency vacuum upon displacement of the denture as in the case of that afforded where peripheral construction and valve seal are complete and perfect. This class of cases may be properly subclassed and would come under the subclass of the *Supplee* class 1 jaws.

### TRIMMING THE LOWER TRAY

The general preparation of the lower tray is the same as that of the upper. The lower jaw, however, has its individual characteristics. One is that absorption takes place in such manner that the curve or circumference of the ridge remains practically unchanged or fixed. Whereas, in the case of the ridge of the maxilla, absorption reduces its circumference quite extensively. Consequently facial contour is less disturbed in the loss of the lower teeth than of the upper. Therefore, less fullness is required in the lower denture for the restoration of disturbed facial contour than in the upper.

The lower jaw, like the upper, also has much overlooked and neglected valuable surface and flexible peripheral tissue surface for base and peripheral adaptation for denture retention. Aside from our general failure to utilize the available area, the lower, like the upper jaw, has as a general rule, two spaces that are much overlooked. These may be called the lingual spaces. They lie on either side of the tongue and are bound by the mylohyoid ridge, the floor of the mouth and the tongue. These spaces are, when present, and utilized, valuable aids to denture retention in those excessively absorbed cases, or so-called flat jaws. Fit the tray well into these spaces, aiming to utilize them in the completed denture. The supporting foundation or ridge of the lower jaw is more or less circular back to the region of the first molars. The diverging flanges formed by carrying the base of the denture into these lingual cavities will act as tangents to the circle and prevent or assist in preventing, horizontal movement of the circular base.

The individual tray being approximately outlined, is now ready for final shaping and conformation to the tissues.

### SHAPING AND CONFORMING THE INDIVIDUAL TRAY

Shape and conform the tray to the tissues without undue pressure or fullness, but under just that degree of pressure and fullness as may be indicated and desired. There are ways of best conforming the periphery of the tray to the flexible tissues. One method is that of the late Dr. Greene, previously referred to, and is no doubt familiar to most of you. The method consists of heating the edge of the black compound tray, and when necessary, tracing modeling compound upon the edge of the tray (tray of Impression Tray Compound the same as upon the edge of a metal tray) and while hot and plastic, inserting the tray in the mouth and having the patient make movements of the muscles which in turn causes the softened tray edge to flow and conform to the tissues.

The other method in its application to the upper jaw consists of successive layers of very thin plaster. As a rule only two mixes are necessary. The first mix registers the position and approximate extent of the imperfections of the improvised compound tray. Where the tray is too long or impinges, the plaster is displaced, and where too short, plaster is added. The tray is freed of excess and points of impingement are cut away to free the tissues impinged. The tray made up partly of tray compound and partly of plaster may now be considered perfect and is ready for the second mix of plaster with which we plan to secure an accurate impression of the jaw under such displacement and pressure upon the tissues as previously decided advisable and predetermined in the preparation of the individual tray.

Since adaptation under pressure upon the flexible tissues of the soft palate is a prerequisite to retention in its maximum degree, and since there is no means of confining or restricting the escape of the flowing thin plaster from the tray about the tissues in this region, as in the case of the labial and buccal borders where the tray is overlapped and bound in by the tissues of the cheeks and lip, it is obvious that adaptation of the palatal border of the denture under pressure upon the soft tissues must, if accurately made, be secured by means of some plastic material, the flowing stress of which offers such resistance as may be required to give the desired pressure upon the tissues.

Modeling compound seems to be the ideal material for use in this connection.

The Foveola Palatine indicate the junction of the hard with the soft palate in the median or to the palatal suture. The Foveola Palatine and the general demarcation between the hard and the soft palate are more accurately outlined in the thin plaster impression just described than with any other method with which we are familiar.

These indications, together with those distinguishing the tuberosities, are taken as guides by which the outline of the soft palate and the length of the base of the proposed denture may be definitely determined.

The posterior border or length of the impression and tray are cut off, conforming their outline to that of the junction of the hard with the soft palate, trimming them to such length as it is desired the finished denture should be.

The remaining outlined plaster representing the impressed surface of the tissues of the soft palate and the extent of this area it is decided the base of the proposed denture should cover, and which it is also desired adaptation of the same should be under pressure, is next entirely cut away and the black compound of the individual tray exposed.

Modeling compound, preferably Kerr's in stick form, is softened with dry heat and traced upon the top of the exposed projecting surface of the black tray. The impression is next dipped into warm water to saturate the plaster and prevent the compound sticking to the tissues when it is inserted into the mouth and adjusted to place. Adjustment of the impression to its seat is made as the varying temperature and flowing resistance of the compound against the tissues being impressed may indicate, to effect the required amount of pressure upon the flexible peripheral tissues of the soft palate as adjustment of the impression to its seat progresses.

Securing adaptation under pressure upon the tissues of the flexible, soft palate by this or some equally scientific means, insuring tension of the tissues under equalized pressure is strongly advocated.

Pressure engagement of the periphery of the denture with the tissues by molding or swaging the base upon casts that have been altered by cutting and scraping to increase the extent of engagement of the periphery with the flexible peripheral tissues, is guesswork and unscientific. The frequent injury of the tissues, and the suffering imposed by such practice, evidence the empiricism of the method and warrant discouragement of its practice. Casts made from accurate impressions secured in accordance with the demands of the case require no cutting or scraping.

Careful study and outline of the hard palate should be made and generous relief of any pressure of the base of the proposed denture upon this area should be certain. Otherwise, pressure of the base upon the tissues of the hard unyielding area may establish a fulcrum, cause rocking of the base and impair the stability of the structure.

### ANATOMICAL OCCLUSION AND ARTICULATION

My understanding of the meaning of the term anatomical occlusion and articulation is, that the teeth, during movements of the mandible, when in the varying positions in the process of mastication, should be so arranged that the cusps of the teeth of the opposing jaws shall establish such relations of contact between them, while gliding to the position of fixed occlusion that varying spaces should be formed to cut, crush and grind the food as the closure of the teeth progresses, providing, thereby, points of support aiding in retention of the dentures, by contact of the antagonizing cusps, with spaces for food.

If this be true, I want to go down in the record as saying that, in my opinion, we have had up to this time, no anatomical occlusion and articulation.

The use of the face bow; the measuring and recording of the condyle paths; the movements of the so-called anatomical articulators; the setting of teeth to theoretically determined planes and curve in wax to the arrangement known as three-point contact, have all been productive of nothing in assimilating anatomical occlusion and articulation. Therefore, no aid to denture retention through this supposed source has been an actual reality, and those advocates and followers of such theories and practices have, in my opinion, been running wild on a delusive theory.

These theories, in part, teach that the general plane of the teeth should be arranged, irrespective of the alveolar ridges, parallel with a line drawn from the ala of the nose to the tragus of the ear. This and its associate rules have long since been found wanting and have not been, nor to my mind will they ever be generally accepted. Although, from the voluminous literature upon the subject, and especially the work and claims of one of our manufacturers, one would be led to believe that the last word on the subject had been said and perfection of its practice attained. Few there are, comparatively, however, that have adopted and are practicing its teachings, and these few will, in time, like the rest of us, discover its shortcomings and abandon its practice. We shall share the keenest pleasure in the disappearance from our text books and literature of these much overworked theories, that we may not be further misguided into error.

Do not be discouraged, however, for there is such a thing as anatomical occlusion and articulation, and when such form and relations of the teeth are secured, not only greater efficiency, but extremely valuable aid to denture retention results. It is my opinion that the form and arrangement of the teeth in jaws greatly resorbed are as important and share equally in importance with that of the construction and adaption of the base and periphery of the denture.

What, then, is anatomical occlusion and articulation? How, and by what means, may we arrange teeth anatomically, so as to secure greater efficiency and their aid in the retention of artificial dentures?

Anatomical occlusion and articulation means that the artificial teeth should be so formed and arranged that they will occlude and articulate without undue interferences, aid in retaining the dentures under the stress of the forces of incision and mastication, guide the co-ordinate muscles in directing the movements of the mandible and at the same time, in the positions of relations of articulation, provide spaces for food.

Teeth may be anatomically arranged by having an articulator capable of imitating all of the movements of incision and mastication of the mandible and then have these movements under such control that we may utilize them for the perfect positioning and grinding of the teeth in anatomical relations as guided by the esthetics, the alveolar ridges and the movements of the articulator.

Present teachings and practices are now wrong. First, because of faulty methods and articulators. Second, because the cusps of the teeth now upon the market are not long enough to permit meshing or interlocking so that in movements of the mandible they will reach and maintain contacts with their antagonists, provide adequate spaces for food and balance the dentures under the stress of the forces of mastication.

Teeth with deep angular cusps (guiding angle 45 degree), correctly arranged, balance the vertical and lateral or horizontal movements of the mandible, as typical to nature, when in the stage of greatest efficiency; definitely guide the co-ordinating muscles in directing the movements of the mandible, also typical to nature; provide spaces for food; balance and support the dentures in the process of incision and mastication; and in case of dislodgement of the dentures, steady and guide their return to their normal position comparable with the guiding and supporting influence of the rails of the railway track upon the coach, the rails, the lower teeth (high cusps), and the wheels, (their flanges, long cusps), the upper teeth. The train without the flanges upon its wheels to secure and guide the movements upon the rails (movements of the mandible in the case of the masticatory apparatus) would leave the track, not travel its intended course and be a failure. Likewise we must justly and logically regard the principles involving the construction and uses of artificial dentures if we are to expect them to work to perform the function of incision and mastication efficiently, and not be a failure.

Especially restorations required in extreme resorbed jaws: We must, therefore, incorporate or provide in their construction, suitably formed and correctly arranged teeth or they will not be guided and supported in the functionating movements of the mandible and will be a failure to such degree as they may be deficient in the requisite fundamentals to the highest and most efficient type of denture restoration.

### FORM AND ARRANGEMENT OF THE TEETH AS AN AID TO DENTURE RETENTION

The present rule is to set the teeth to approximate a length in line with the lips and not overlap the upper or under-bite the lower anterior teeth to any considerable degree, owing to leverages that would be formed, dislodge the dentures and break off the anterior teeth.

This would be a very fine rule if turned the other way round. The rule is absolutely wrong and its application is the greatest cause for dislodgement of dentures and breakage of anterior teeth. Why? Because, to set the anterior teeth in line with the lips and not permit them, when occluded, to pass or overlap as in nature, means simply that the molars and bicuspids have to be lengthened to a bite corresponding in distance between the ridges with the incisive bite, instead of the occluded bite. In other words, following this rule produces dentures, the presence and operation of which in the mouth force the bite open to the extent of the depth of normal overbite, and interferes with the movements of the mandible, and the patient experiences a feeling that the mouth is held open and the teeth are too long. Therefore, teeth so arranged do not efficiently restore the function of incision and mastication, and the excessively long molars and bicuspids interfere in the movements of incision and mastication, and impair retention of the dentures.

In the absence of overlapping anterior teeth, as normal to nature and necessary to the functions of incision, the patient manages to find a point of contact between two of the opposing end to end related incisors and in their effort to incise it is discovered that the teeth do not slide by and shear the object attempted to be incised, but merely pinch or puncture it, and in this predicament the patient thoughtlessly, though naturally, pulls the object in two, with the frequent result of a broken tooth.

The incisors, if correctly arranged to overlap, and ground to establish perfect opposing articulating edges and planes, will, with the minimum amount of pressure, efficiently perform the function of incision and equally distribute the pressure upon all the anterior teeth. And in so arranging them the molars and bicuspids will be relatively shortened and normal anatomical relations be restored.

Teeth should be so arranged that they conform to the esthetic, anatomical and physiological needs of the patient.

The misleading and absurd rule that confines us to a definite line of procedure in obtaining the relation the teeth and jaws should bear to each other, irrespective of the modifying influences of the individual patient, should, in my opinion, be disregarded altogether.

Each case of denture construction is a law unto itself and no rule of averages, be it ever so inclusive, can possibly supplant or adequately supply the specific demands arising in the individual case. The rule that the length of the teeth should be established in accordance with the lips when in position of repose, is no less impractical than is the rule of establishing the occlusal plane.

The misleading feature of this rule, by which we are supposed to determine the length of the teeth, is due entirely to the fact that the rule does not conform to the conditions imposed in nature. When the muscles controlling mandibular movements are in a state of rest the lips normally are extended to their full length. The muscles controlling mandibular movements are not constantly contracted—nature could not tolerate a constant contraction of these muscles—and in their unfunctionating state the mandible hangs down to the extent of relaxation of the suspending muscles and tendons and the teeth are not normally in occlusion. The reverse of this normal repose in lip position would be found when the teeth are occluded and the lips are compressed and much shortened. It is my idea that the latter position of the lips more nearly indicates the length of the bite when the teeth are in occlusion than does the position of the lips when the muscles are relaxed and at rest.

I have dealt at length on this particular point because it will help to emphasize the things I am contending are wrong in respect to some of the rules and theories heretofore set down as definite facts, which in my judgment, are very great hindrances to our success and advancement in the art of denture construction, both as regards retention and efficiency.

